# Successful Treatment with Mycophenolate Mofetil and Tacrolimus in Juvenile Severe Lupus Nephritis

**DOI:** 10.1155/2015/651803

**Published:** 2015-02-17

**Authors:** Tomoo Kise, Hiroshi Yoshimura, Shigeru Fukuyama, Masatsugu Uehara

**Affiliations:** Division of Pediatric Nephrology, Okinawa Prefectural Nanbu Medical Center-Children's Medical Center, Arakawa 118-1, Haebaru, Okinawa 901-1193, Japan

## Abstract

Lupus nephritis (LN) of juvenile onset often has severe disease presentation. Despite aggressive induction therapy, up to 20% of patients with LN are resistant to initial therapy and up to 44% suffer a renal relapse. However, there is no consensus on an appropriate therapeutic regimen for refractory LN. We report a 13-year-old girl with recurrent LN who was not taking her medications. At age of 11 years, she was diagnosed with LN classified as International Society of Nephrology/Renal Pathology Society (ISN/RPS) class IV G (A) + V. She was treated with prednisolone and MMF after nine methylprednisolone pulses. Nineteen months later, she was admitted to the hospital with generalized edema. Her symptoms were nephrotic syndrome and acute renal dysfunction. She received three methylprednisolone pulses for 3 days, followed by oral prednisolone and MMF. Twenty-seven days after the three methylprednisolone pulses, her acute renal dysfunction was improved, but the nephrotic syndrome was not improved. A second biopsy showed diffuse lupus nephritis classified as the predominant finding of ISN/RPS class V. We added tacrolimus to the MMF. Four months after adding tacrolimus, the nephrotic syndrome improved. We conclude that adding tacrolimus to the treatment regimen for LN resistant to MMF is effective.

## 1. Introduction

Juvenile onset lupus nephritis (LN) often has very active and severe disease presentation. Several studies have shown that mycophenolate mofetil (MMF) is at least as effective as intravenous cyclophosphamide (IVCY) for active LN [1–3]. Despite aggressive induction therapy, up to 20% of patients with LN are resistant to initial therapy and up to 44% suffer a renal relapse [4]. However, there is no consensus on an appropriate therapeutic regimen for refractory LN.

Tacrolimus (Tac) is an immunosuppressive macrolide of the calcineurin inhibitors. Recently, the addition of Tac to treatment with MMF has been reported to be useful in refractory LN [5, 6]. We report a 13-year-old girl with recurrent LN (nephrotic syndrome and acute renal dysfunction), who was not taking her medications. Administration of three methylprednisolone pulses and doubling of the MMF dose improved the acute renal dysfunction but could not improve the patient's nephrotic syndrome. Tacrolimus was introduced to control disease activity.

## 2. Case Report

**Figure 1:**
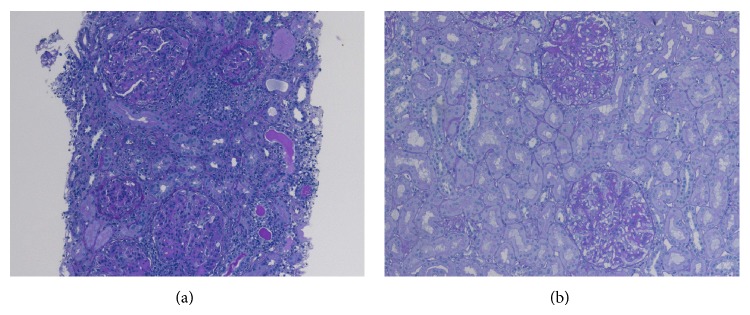
Renal biopsy findings. (a) Initial renal biopsy findings. Light microscopy reveals diffuse global mesangial proliferation. Renal interstitium is filtrated by lymphocytes. Tubular atrophy and tortuosity are shown. Periodic acid-Schiff (PAS) staining, ×200. (b) Second renal biopsy findings. Light microscopy revealed that the mesangial proliferation and the renal tubular lymphocyte infiltration are improved. Tubular atrophy and tortuosity are not shown. PAS staining, ×200.

A 13-year-old girl was admitted to our hospital emergently with anasarca and renal dysfunction. At the age of 11 years, she was diagnosed with systemic lupus erythematosus (SLE) according to the American Rheumatism Association criteria, based on positive antinuclear antibody, positive anti-double stranded DNA (dsDNA) antibody, pancytopenia, and persistent proteinuria. Renal dysfunction was showed in serum creatinine of 0.8 mg/dL, blood urea nitrogen of 28 mg/dL, and an estimated glomerular filtration rate (eGFR) of 80 mL/min/1.73 m^2^ using the Schwartz formula. Urine protein creatinine ratio was 9.01 g/g Cr. Urinary sediment showed 30–49 red blood cells/high power field (HPF), 5–9 white blood cells/HPF, hyaline cast, and granular cast. Renal biopsy showed diffused lupus nephritis classified as International Society of Nephrology/Renal Pathology Society (ISN/RPS) class IV G (A) + V associated with cellular crescents (60%), endocapillary hypercellularity, karyorrhexis, wire loop lesions, leukocyte infiltrates, and subepithelial immune deposits separated by spikes (Figure 1(a)). Immunofluorescence revealed “full-house” staining for C3, C1q, IgG, IgA, and IgM in the mesangial and endocapillary regions. She was treated with prednisolone at 30 mg/day (0.75 mg/kg/day, body weight 40.3 kg) and MMF at 1 g/day after nine methylprednisolone pulses (1000 mg/day) for 18 days. At 7 months after being diagnosed with LN, she became well, and prednisolone was reduced to 10 mg/day while maintaining MMF at 1 g/day. She had lost 2 kg in weight and her edema was relieved. Her urine protein creatinine ratio had decreased to 6 g/g Cr, and her serum albumin had increased to 2.6 g/dL.

Six months later, she was admitted to the hospital with generalized edema. At admission, she was found to have gained 8 kg in weight over the last three weeks. Vital signs were stable except for mild hypertension with blood pressure of 126/78 mmHg. There was the absence of extrarenal disease. Laboratory studies (Table 1) at admission showed hypoproteinemia (4.0 g/dL), hypoalbuminemia (1.5 g/dL), renal dysfunction (blood urea nitrogen 12 mg/dL, serum creatinine 0.9 mg/dL, and eGFR 74 mL/min/1.73 m^2^), and hypocomplementemia (C3 50 mg/dL). Hemoglobin was 12.5 g/dL. The early morning urine-protein to creatinine ratio was 7.18 g/g Cr. Urinary sediment showed 5–9 red blood cells/HPF, 10–19 white blood cells/HPF, granular cast, and oval fat body. She admitted that she had not been taking her medicine. The patient received three methylprednisolone pulses (500 mg/day) for 3 days, followed by oral prednisolone increased to 30 mg/day from 10 mg/day, and MMF was increased to 2 g/day from 1 g/day. In addition to benzylhydrochlorothiazide, we administered furosemide at 40 mg/day to improve the anasarca. One month later, after administration of the three methylprednisolone pulses, the patient's serum creatinine improved to 0.5 mg/dL; eGFR was 116 mL/min/1.73 m^2^. Her acute renal dysfunction was improved, but her nephrotic syndrome became worse (Table 1). Serum albumin was 1.8 g/dL, and immunoglobulin G decreased from 540 mg/dL on admission to 130 mg/dL. Hemoglobin was 13.6 g/dL. Urine-protein to creatinine ratio was 7.97 g/g Cr. Urinary sediment showed 5–9 red blood cells/HPF, 10–19 white blood cells/HPF, granular cast, and oval fat body. We had to replenish gamma globulin. On the following day, a second renal biopsy was performed. Light microscopic examination showed 51 glomeruli, of which 3 were globally sclerotic. The remaining 48 glomeruli had global subepithelial spike formation (Figure 1(b)). Wire loop lesions, mesangial cell proliferation, karyorrhexis, hematoxylin bodies, and cellular crescents could not be seen in any glomeruli. Neither lymphocyte infiltration nor neutrophils were apparent in the glomeruli or interstitium. Immunofluorescence revealed staining for C3, C1q, IgG, and IgA in the mesangial and endocapillary regions. On the basis of these findings, we made a diagnosis of lupus nephritis classified as a predominant finding of ISN/RPS class V. Based on active renal lesions seen in the second biopsy, we administered tacrolimus (3 mg/day) in addition to oral prednisolone and MMF. We titrated the dose so that the tacrolimus blood concentration increased to 5 ng/mL. One month after the addition of tacrolimus, it was no longer necessary to replenish gamma globulin for augmentation of immunoglobulin G. By the end of 4 months of tacrolimus therapy, all laboratory findings and the patient's anasarca were improved (Table 1). We explained to the patient and her father the need for taking the medicine, and she was discharged.

Three months after discharge, she was free of edema, and her blood concentration of tacrolimus was maintained. In the outpatient department, she reported that she was “taking the medicine well.”

## 3. Discussion

Adding tacrolimus to MMF and prednisolone in this patient with severe LN was effective. Bao et al. reported the effectiveness of the combination of MMF, tacrolimus, and prednisolone (multitarget therapy) as induction therapy for lupus nephritis [6]. Cortés-Hernández et al. and Lanata et al. reported that adding tacrolimus for MMF-resistant LN patients (classes III, IV, and V) was effective [4, 5]. Mok et al. reported that they administered triple-drug therapy of MMF, tacrolimus, and prednisolone to lupus nephritis patients for whom a regimen of two immunosuppressive regimens had been insufficient; the urine protein was improved in 75% of the patients [7].

The treatment of the MMF-resistant lupus nephritis include the multitarget therapy and various biologics that target B cells, T cells, or different cytokines [8]. Cyclophosphamide or cyclosporine is considered as treatment of class V LN with first choice [9]. On the other hand, for the treatment of class IV LN, cyclophosphamide or MMF is first choice. MMF is the prodrug of mycophenolic acid (MPA), which blocks antibody formation by polyclonally activated B-lymphocytes. MPA also blocks production of cytokines such as interleukin- (IL-) 2, IL-10, interferon- (IFN-) *γ*, and tumor necrosis factor-alpha (TNF-*α*) [7]. Tacrolimus blocks the phosphate activity of calcineurin and reduces IL-2 gene transcription of activated T cells. Blocking T cell activation also leads to reduction in the production of other proinflammatory cytokines that are important in regulating B cell activity [7]. These authors explained that, because the mechanism of action was different using tacrolimus, MMF, and steroids, the combination of these three drugs could control inflammatory, proliferative, vasculitic, and membranous lesions synchronously [6, 7].

The membranoproliferative class diagnosed in the initial biopsy was transformed to membranous lupus nephritis despite immunosuppressive treatment with MMF. The multitarget therapy was effective in our case of class V LN. There are few reports of such transformations. Dalton et al. stated that it was unclear whether treatment of their patient with MMF had contributed to the development of a membranous lesion or it had been inadequate treatment for the lesion as it developed [10].

In our patient, no adverse effects were observed during a treatment period of 6 months. In the four reports mentioned above [4–7], 62 adverse effects were noted in 62 patients: infection not requiring hospitalization, 16 cases; digestive symptoms (diarrhea, dyspepsia, and nausea), 9 cases; major infection requiring hospitalization, 7 cases; muscle symptoms, 6 cases; herpes infection, 5 cases; hypercholesterolemia, 4 cases; hypertension, 3 cases; hyperglycemia or diabetes mellitus, 3 cases; leukopenia, 2 cases; transient increase in serum creatinine, 2 cases; alopecia, 2 cases; tremor, 2 cases; and irregular menstruation, 1 case. None of these adverse effects led to protocol withdrawal. Mok et al. stated that the long-term risk of nephrotoxicity and adverse vascular effects could be minimized by using a lower dose of tacrolimus [7].

In conclusion, we believe that adding tacrolimus to MMF and prednisolone in patients with juvenile onset lupus nephritis is effective. Long-term observation is needed to determine whether this therapy continues to be tolerated and effective.

## Figures and Tables

**Table 1:** Laboratory findings on admission, at 1 month after methylprednisolone pulses and at discharge.

	At admission	1 month from methylprednisolone pulses	1 month from addition of tacrolimus	At discharge (4 months from addition of tacrolimus)
White blood count (×10^2^/*μ*L)	51	193	85	82
Hemoglobin (g/dL)	12.5	13.6	10.2	11.5
Platelets (×10^4^/*μ*L)	30.9	37.3	28	29.9
Total protein (g/dL)	4.0	3.9	4.6	5.5
Albumin (g/dL)	1.5	1.8	2.4	3.0
Blood urea nitrogen (mg/dL)	12	18	18	12
Creatinine (mg/dL)	0.9	0.5	0.5	0.5
C3 (mg/dL)	50	63	88	111
Immunoglobin G (mg/dL)	540	130	218	371
dsDNA antibodies (IU/mL)	98	15	14	12
Urine-protein to creatinine ratio (g/gCr)	3.13	7.97	2.79	1.07
Urine sediment				
RBC/HPF	5–9	5–9	<1	<1
WBC/HPF	10–19	10–19	1–4	1–4
Cast	Granular (+)	Granular (+)	(−)	(−)
Oval fat body	(+)	(+)	(+)	(−)
